# Innovative One-Step Sustainable Process to Produce Simonkolleite Nanoparticles

**DOI:** 10.3390/nano14242005

**Published:** 2024-12-13

**Authors:** Valeria Daniele, Claudia Mondelli, Laura Turilli, Giuliana Taglieri

**Affiliations:** 1 Department of Industrial and Information Engineering and Economics, University of L’Aquila, Piazzale E. Pontieri 1, Monteluco di Roio, Roio Poggio, 67100 L’Aquila, AQ, Italy; laura.turilli@graduate.univaq.it; 2 CNR-IOM-OGG, Institut Laue Langevin, 71 Avenue des Martyrs, CEDEX 9, 38042 Grenoble, France; mondelli@ill.fr

**Keywords:** sustainable and scalable synthesis, simonkolleite nanoparticles, XRD, TEM, FTIR, BET

## Abstract

The aim of the present paper is to propose an innovative, one-step and sustainable process allowing us to obtain almost 10 kg/week of pure and crystalline simonkolleite nanoparticles (SK NPs) in only 8 min of reaction, working in water, under ambient conditions of pressure/temperature, guaranteeing at the same time low environmental impact and a high yield of NP production. In addition, the obtained NPs can also act as ZnO precursors at ambient temperature, and this result supports the sustainability of the process considering that, generally, the production of ZnO from SK occurred via annealing at high temperatures. The SK NPs appeared pure and crystalline, characterized by a highly uniform hexagonal lamellar feature. Each lamella is composed of an ordered assembly of very small monodispersed primary NPs, with a size in the range 3–8 nm. The SK NPs exhibited a surface area of up to 41 m^2^/g, the highest value recorded in the literature, revealing that pore size distribution mainly peaked between 3 and 20 nm.

## 1. Introduction

Simonkolleite, a zinc chloride hydroxide monohydrate (Zn_5_(OH)_8_Cl_2_·H_2_O), is a layered double hydroxide salt, crystalizing in the hexagonal system and having an excellent cleavage on the {001} plane [1], and it is characterized by a brucite-like structure [2,3,4]. Due to the presence of flat layers, simonkolleite generally appears as a 2D nanosheet, characterized by micro platelet crystals with hexagonal morphology [3,5,6,7]. The two-dimensional layers are composed of ZnO_6_ octahedra interconnected with ZnO_3_Cl tetrahedra, arranged across the *c* axis in a layered 3D structure, with the water molecules intercalated between the layers and Cl atoms preferentially directed toward the same interlayer space [3,4,8,9]. The availability of oxygen vacancies on the surface makes the simonkolleite electrically and chemically active [10,11,12,13], because these vacancies can act as n-type donors, improving the material’s conductivity and making the simonkolleite suitable for efficient charge storage. In addition, for supercapacitor applications, the layered structure, composed of a hexagonal platelet interactive network, can provide the surface required for ionic surface interactions during operation [12]. Simonkolleite can also be used for many other applications, such as water pollutant removal agents [14], catalysts [15], gas sensors [11,16], UV and visible light absorbents [17,18], flame retardants [19,20], nanofillers [21,22], feed additives and nutritional supplements for animals [23], anticorrosion agents [24,25], and drug carriers in systems requiring slow release [26]. In addition, considering both the chemical composition of simonkolleite (which is a hydroxide salt) and its ability to transform into ZnO, this compound has recently garnered attention, particularly in nanostructured form, in medical applications as an antibacterial agent, showing good properties in the healing of deep wounds in a moist environment [27,28,29]. Finally, the ability of the simonkolleite nanostructure to slowly release bioactive Zn ions as part of bioprocesses can be considered an auspicious achievement for the improvement in bio methanation and CH_4_ production rate [15]. Based on all its potential applications, simonkolleite represents a versatile technological material, and gaining a full understanding of its properties constitutes a key point for future extension of its industrial implementation.

Regarding its availability, currently, several synthetic routes for synthesizing pure simonkolleite with a specific nanostructure under various conditions are employed, often consisting of hydrolysis of divalent metal salts in the presence of a metal oxide [30,31], interdiffusion of ammonia in aqueous media [32], using the precipitation method by adjusting the pH to prevent competing reactions, resulting in the formation of Zn(OH)_2_ [4,33], using the sol–gel hydrothermal technique under acidic conditions [15], and using hydrothermal methods [23]. Nevertheless, most of these processing approaches are typically expensive and time-consuming, as well as potentially harmful to the environment, lacking the fundamental aspect of sustainability in relation to the use of volatile and corrosive conventional solvents (i.e., ammonia), the non-environmental working conditions, the production of damaging wastes, and the necessity to adjust the pH of the solution to prevent undesired reactions. In particular, the processes are often slow, going through multiple intermediate products and thus negatively influencing the yield of production [4,8] and detracting from the potential use of simonkolleite nanoparticles in large-scale applications. As a result, a cost-effective, safe, and environmentally friendly method to produce simonkolleite nanoparticles is even more necessary. For this task, at the University of L’Aquila, we developed an innovative, eco-friendly, and sustainable synthetic route to obtain in only 8 min almost 10 kg/week of pure and crystalline simonkolleite nanoparticles, paving the way for the scalability of its production to commercial applications. The proposed approach, based on an ion exchange process and already patented for the production of other metal oxide/hydroxide NPs [34,35], allows us to produce in a fast way pure and crystalline simonkolleite nanoparticles, operating under environmental working conditions (ambient temperature/pressure), in a single step, with low energy consumption, by using renewable reagents and without producing any damaging waste. The employed resin, separated from the suspension at the end of the process, can be regenerated and immediately reused, leading to a cyclic procedure [36].

The aim of the present paper is to study the kinetics of simonkolleite formation as well as the phase purity of the obtained product, by means of X-ray powder diffraction (XRD), by varying both the initial reagent ratio and the synthesis temperature. In addition, more detailed studies regarding particle dimensions and morphologies were performed by using transmission electron microscopy (TEM), while surface area measurements were carried out on the suspension, giving rise to the best results in terms of the residual chloride content and production yield.

## 2. Materials and Methods

### 2.1. Materials

Zinc chloride (ZnCl_2_), characterized by a purity >98%, was provided by Sigma Aldrich; the ion exchange resin Dowex Monosphere 550A was supplied by Sigma Aldrich (St. Louis, MO, USA) and it is composed of translucent spherical beads with a particle size of 590 ± 50 µm. Sodium hydroxide (NaOH) pellets, with a purity of 98%, were supplied by Sigma Aldrich.

### 2.2. Synthesis of Simonkolleite Nanoparticles

Following the recommendations reported in our previous paper [36], the simonkolleite nanoparticles (SK NPs)—in the form of an aqueous suspension—were synthesized by means of our revolutionary procedure, already patented for the production of different metal oxide/hydroxide NPs [34,37].

Working at room temperature (T = 25 °C), a 1 M ZnCl_2_ aqueous solution, employed as the initial reagent, was put in contact under moderate stirring and for only 8 min with a proper amount of anionic resin in OH^−^ form, reaching a total volume of 400 mL [34]. During the reaction, the Cl^−^ ions present in the aqueous solution were quickly transferred to the resin, which exchanged its OH^−^ ions into the water. At the end of the synthesis, the aqueous dispersion of SK NPs was separated from the resin by means of a sieving procedure.

During the stirring operation, the kinetics of the ion exchange process was determined by taking homogeneous samples from the suspension at different reaction times (t = 0, 0.5, 1, 2, 3, 4, 5, 6, 7, and 8 min, respectively). After the withdrawal, performed by using a wide opening tip, we immediately separated the resin from the suspension, and the collected samples were subjected to chloride concentration measurements and XRD analyses. In particular, the variation in the chloride’s concentration (*CC*), from the beginning to the end of the synthesis, was measured by means of an ion-sensitive electrode (Metrohm, Switzerland), allowing us to define the yield of the ion exchange process (*Y*), according to the formula reported below:

(1) $$Y\left(t\right)=\left(\frac{{CC}_{0}-CC(t)}{C_{0}}\right)*100$$

where *CC*_0_ is the chloride concentration at time *t* = 0, while *CC*(*t*) represents the chloride ion concentration value at time *t*.

Following the same procedures previously described, several syntheses were performed by varying the resin/ZnCl_2_ (*R*) ratio (*w*/*w*) to investigate the influence of the *R* ratio itself on the kinetics of simonkolleite. In particular, five resin/ZnCl_2_ ratios were considered, corresponding to 0.8:1 (*0.8R* sample), 1:1 (*1R* sample), 1.5:1 (*1.5R* sample), 2:1 (2*R* sample), and 3:1 (*3R* sample), respectively.

Moreover, by using the “optimal” *R* ratio, both in terms of Cl^−^ content reduction and production yield, we carried out another 3 syntheses by varying the temperature conditions (equal to T = 8 °C, 18 °C, and 45 °C) to analyze the influence of the temperature itself on the kinetics of simonkolleite.

### 2.3. Characterization of Synthesized Simonkolleite Nanoparticles

The phase purity and crystallinity of all of the produced aqueous suspensions were analyzed by means of the XRD technique; we carried out this procedure on the powders dried at ambient temperature (T = 25 °C). The XRD spectra were recorded on a PANalytical X’PertPRO apparatus (Almelo, The Netherlands) using CuKα radiation, with a step scan covering the angular range 2θ from 5° to 60° and a step size 2θ = 0.026°. Then, the experimental diffraction patterns were elaborated by means of Profile Fit Software (HighScorePlus 4.9 software package, PANalytical), and the crystalline phases were determined according to ICDD and ICSD international reference databases.

ATR-FTIR measurement of the simonkolleite NPs was performed by using a NexusTM 870 FT-IR (Thermo Fisher Scientific, Waltham, MA, USA) spectrophotometer over a range of 400–4000 cm^−1^ at a resolution of 4 cm^−1^. The ATR spectrum of a thin pellet of nanoparticles, in form of oven-dried (T = 50 °C) powder, in a KBr matrix was analyzed.

Particle dimensions and morphologies were investigated on the samples coming from the syntheses performed by using the best *R* ratio but varying the temperature conditions. In particular, the particles’ morphology was analyzed by means of transmission electron microscopy (TEM, Philips CM100), according to standard procedures, while the particles’ size distribution was evaluated by using ImageJ 1.54g software bundled with Java 1.8.0.

Concerning the surface area measurements, N_2_ adsorption analysis was carried out at 77 K with a *Quantachrome Nova system* utilizing the Brunauer–Emmett–Teller (BET) method. Approximately 0.5 g of SK powders, coming from the centrifuged samples dried in an oven at 50° C, was outgassed for about 24 h at 120 °C. In particular, BET analysis provides a precise specific surface area evaluation of materials by means of nitrogen multilayer adsorption measured as a function of relative pressure using a fully automated analyzer. Considering that most gases and solids interact weakly, the solid material must be cooled, typically using a cryogenic liquid. The temperature of the solid sample is kept constant, while the pressure of the adsorbing gas is increased, allowing more and more molecules to adsorb on the surface. The number of gas molecules in the monolayer is recorded from the volume adsorbed. Since the cross-sectional area of the adsorbate is known, the total specific surface area in m^2^/g can be calculated. Finally, the BJH (Barett–Joyner–Halenda) analysis was employed to determine pore area and specific pore volume using adsorption and desorption techniques. This technique characterizes pore size distribution independently of the external area due to the particle size of the sample.

## 3. Results and Discussion

**Figure 1:**
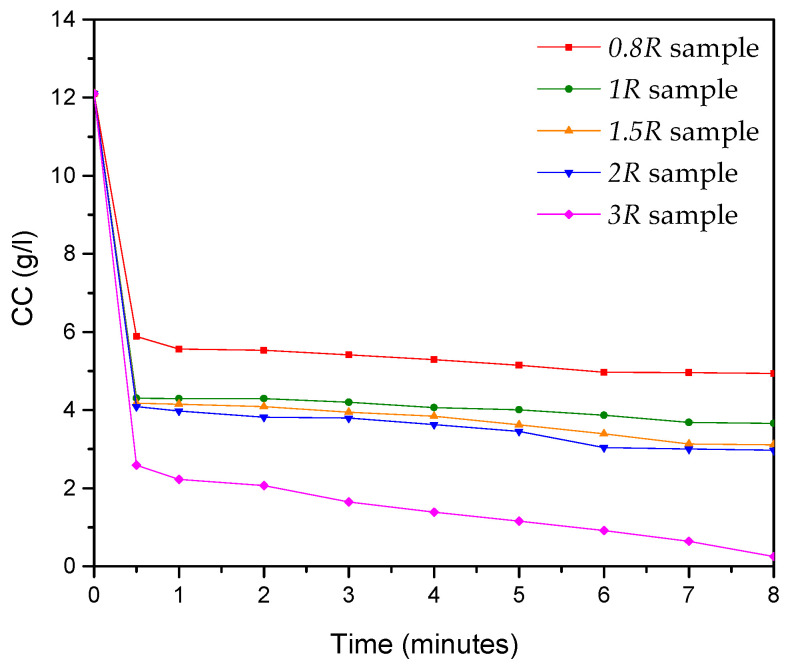
Kinetics of ion exchange process in terms of CC versus time, referring to syntheses performed at ambient temperature (T = 25 °C). Five resin/ZnCl_2_ ratios were considered, corresponding to 0.8:1 (*0.8R* sample), 1:1 (*1R* sample), 1.5:1 (*1.5R* sample), 2:1 (*2R* sample), and 3:1 (3*R* sample).

As reported in Section 2.2, the kinetics of the ion exchange process was analyzed by taking, from the suspension, homogenous samples at different reaction times from the beginning to the end of the synthesis process. The results obtained (Figure 1) show that our synthetic route is characterized by fast reduction in chloride concentration (CC) in the first few seconds, particularly marked for the *3R* sample, with a decrease in CC values ranging from 12.1 g/L (t = 0 min) to 2.59 g/L (t = 0.5 min). After 0.5 min, a near-horizontal behavior is observed, reaching the least saturated value in the *3R* sample and also showing the highest Y (≅98%) among all of the considered samples (Table 1).

**Figure 2:**
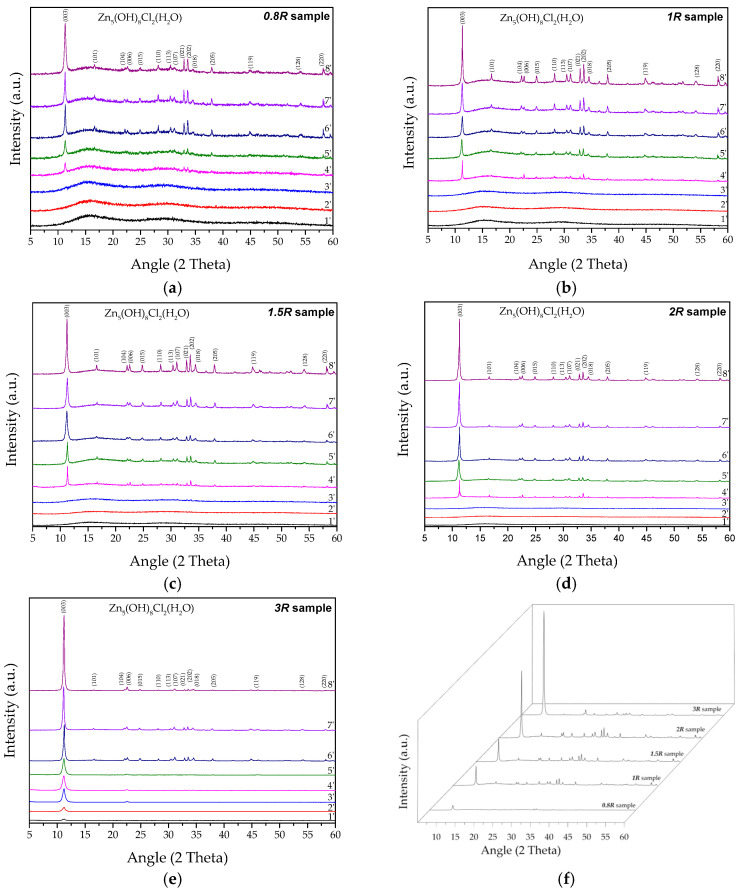
XRD analyses performed at different times from the beginning of the synthesis process by varying the resin/ZnCl_2_ (R) ratios. (**a**) *0.8R* sample; (**b**) *1R* sample; (**c**) *1.5R* sample; (**d**) *2R* sample; (**e**) *3R* sample; (**f**) a comparison between the samples at the end of the synthesis process (t = 8 min) by varying the *R* ratio.

The XRD analyses, reported in Figure 2, allowed us to investigate the time necessary to produce SK considering different *R* ratios. In particular, in the *0.8R*, *1R*, *1.5R*, and *2R* samples, the initial formation of a unique phase attributable to pure SK (Zn_5_Cl_2_(OH)_8_·H_2_O, ICSD #98-003-4904, see Figure S1) can be observed after 4 min of synthesis (Figure 2a–d), and this phase increased by increasing the synthesis time.

Regarding the *3R* sample (Figure 2e), the initial formation of the SK phase begins already after only 1 min, underlining the role of the resin/ZnCl_2_ ratio in promoting SK formation; this result is totally in accordance with the CC reduction and Y values reported in Table 1.

The sharp and strong peaks confirmed the high crystallinity of the product obtained. Moreover, all of the XRD spectra showed that the SK NPs have a strong preferential orientation along the (003) direction, with an angular position of 11.22° 2θ_(003)_, corresponding to the highly ordered reticular planes related to the formation of a simonkolleite processing layered structure [11,29]. As reported in a previous literature paper [11], this growth habit can be originated from the hexagonal facet in nucleation and the subsequent layer growth mode along the (003) plane, underlining the plate-like structure of the synthesized SK. In particular, SK contains brucite-like zinc hydroxide layers, consisting of reticular planes of hexagonally ordered octahedrally coordinated zinc cations and tetrahedrally coordinated zinc cations occupied above and below the vacant octahedral units. These layers, perpendicular to the c-direction, are bridged by chloride anions and water molecules [23].

If synthesis with the *3R* ratio lasts over 8 min, gradual conversion of SK into the ZnO phase would occur at ambient temperature due to the fact that SK is not in a stable phase over time [2,36]. In particular, by increasing the exchange reaction time, the excess of hydroxyl ions in the solution gave rise to an alkaline ambient, favoring the formation of zincate ions (Zn(OH)_4_^2−^) acting as precursors for the nucleation and crystal growth of ZnO at ambient temperature, as also reported in our previous paper [36].

Finally, by overlapping the XRD spectra coming from the samples at the end of the synthesis process (t = 8 min), it was possible to note that SK production was significantly higher when the *3R* ratio was employed (Figure 2f), and these results are totally in agreement with the Y values reported in Table 1.

Considering that the synthesis performed by using the *3R* ratio gave rise to the highest yield of production together with a drastic reduction in the CC values, we fixed this ratio to perform another three syntheses by varying the temperature conditions, allowing us to investigate the influence of temperature itself on the kinetics of SK formation.

**Figure 3:**
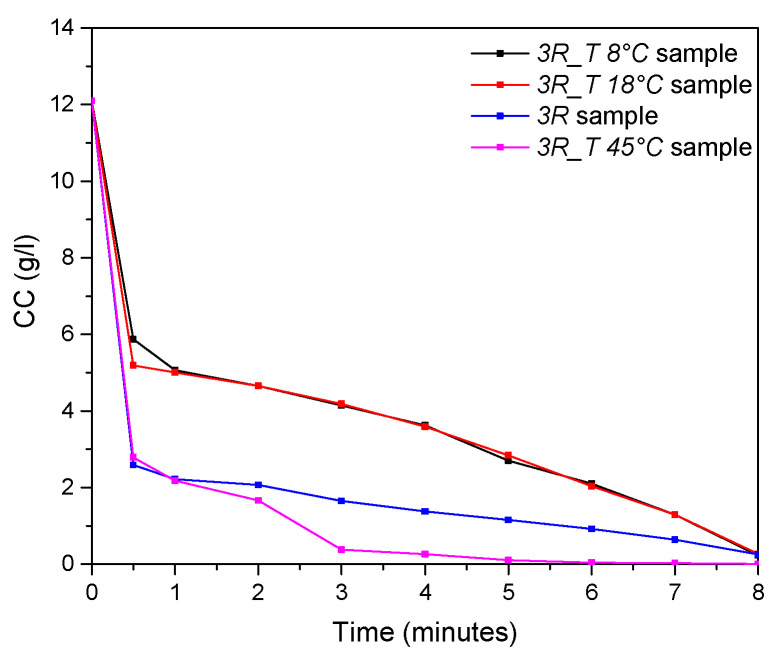
The kinetics of the ion exchange process, in terms of the CC versus time, referring to the syntheses performed with the 3*R* ratio by varying the temperature conditions.

The obtained results, summarized in Figure 3 and Table 2, revealed that, particularly when the highest temperature was employed (*3R_T 45 °C* sample), a drastic reduction in the Cl^−^ content from 12.1 to 0.38 g/L was achieved in the first 3 min of the reaction, leading to a corresponding Y of about 97%.

In the other samples (*3R_T 8 °C* and *3R_T 18 °C*), when the synthesis process was stopped, a clear reduction in the CC content was registered, with values ranging from 12.1 (t = 0 min) up to less than 0.27 g/L (t = 8 min), thus indicating the total transformation of the initial reagent into the final product, with a corresponding Y of about 98%.

**Figure 4:**
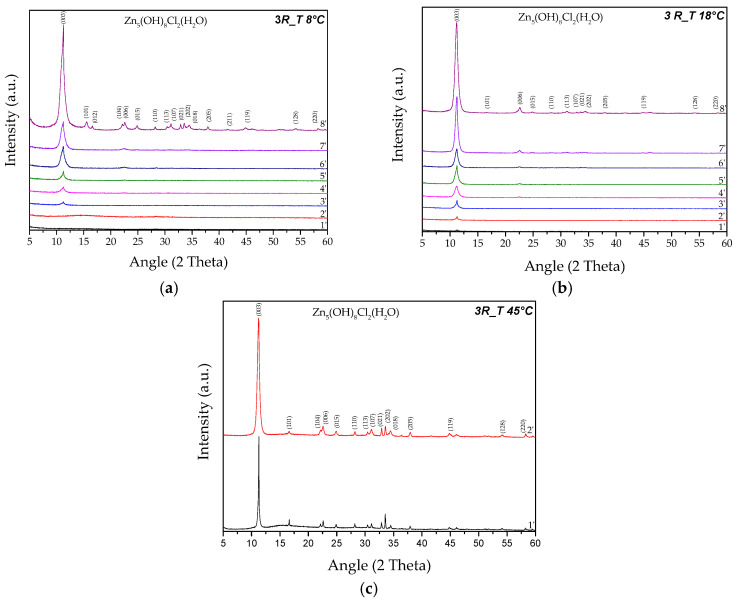
The XRD analyses performed on the samples obtained by using the *3R* ratio but varying the synthesis temperature. (**a**) T = 8 °C (*3R_T 8 °C* sample); (**b**) T = 18 °C (*3R_T 18 °C* sample); (**c**) T = 45 °C (*3R_T 45 °C* sample).

The XRD analyses revealed that when the temperature condition equal to 18 °C was established (*3R_T 18 °C* sample), no marked differences compared to the sample obtained at ambient temperature (*3R* sample) were noted, as shown in Figure 4b and Figure 2e. The first traces of crystalline SK appeared after 1 min of reaction, and this phase remained unique and stable up to a reaction time of 8 min. In contrast, a low synthesis temperature (*3R_T 8 °C* sample) gave rise to slower kinetics of the ion exchange process, showing the initial formation of SK after 3 min from the beginning of the synthesis process (Figure 4a).

Finally, by working with the highest temperature condition (*3R_T 45 °C* sample), the kinetics of SK formation appeared very rapid, underlining the role of temperature in promoting simonkolleite production (Figure 4c). This result confirms both the CC reduction and the corresponding Y values reported in Table 2, leading to the production of a high quantity of pure SK in just 1 min. This phase remains stable up to 2 min of reaction, above which the partial conversion of SK into ZnO can be observed. For this reason, when the T = 45 °C condition was chosen, in order to obtain pure SK, the synthesis must be stopped after 2 min from the beginning of the process.

**Figure 5:**
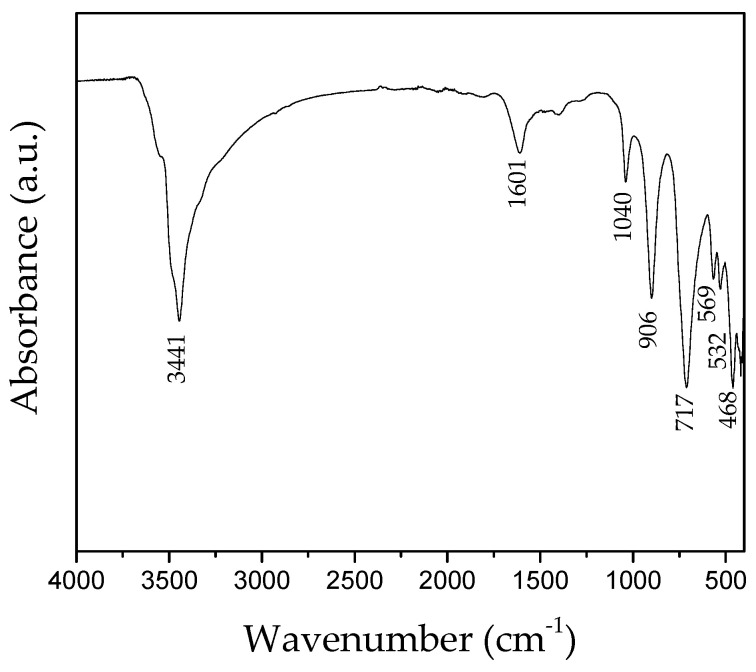
A typical infrared attenuated total reflection spectrum of the synthesized simonkolleite NPs.

To support the XRD results in terms of the purity of the produced SK NPs, infrared attenuated total reflection (ATR-FTIR) analyses were performed. In particular, as shown in Figure 5, a typical ATR-FTIR spectrum, coming from the *3R* sample collected at the end of the synthesis process (t = 8 min), was reported. It is possible to note the presence of a strong band centered at 1601 cm^−1^ due to the deformation vibration of H_2_O molecules, together with an absorption band at 3441 cm^−1^, characteristic of the O-H stretching vibration. As also discussed in the literature [11], hydrogen bonds are formed between the H_2_O molecules (H bond donors) and the OH groups (H bond acceptors) in Zn_5_(OH)_8_Cl_2_·H_2_O. Specific bands at lower wavenumbers are recognized as well, related to various stretching vibration modes of chloride ions. The bands centered at 906 and 717 cm^−1^ are assigned to Zn-OH vibrations and those below 620 cm^−1^ to lattice modes. Finally, the bands at 569, 532, and 468 cm^−1^ are attributed to the translational modes of the Zn-O bonds [11].

**Figure 6:**
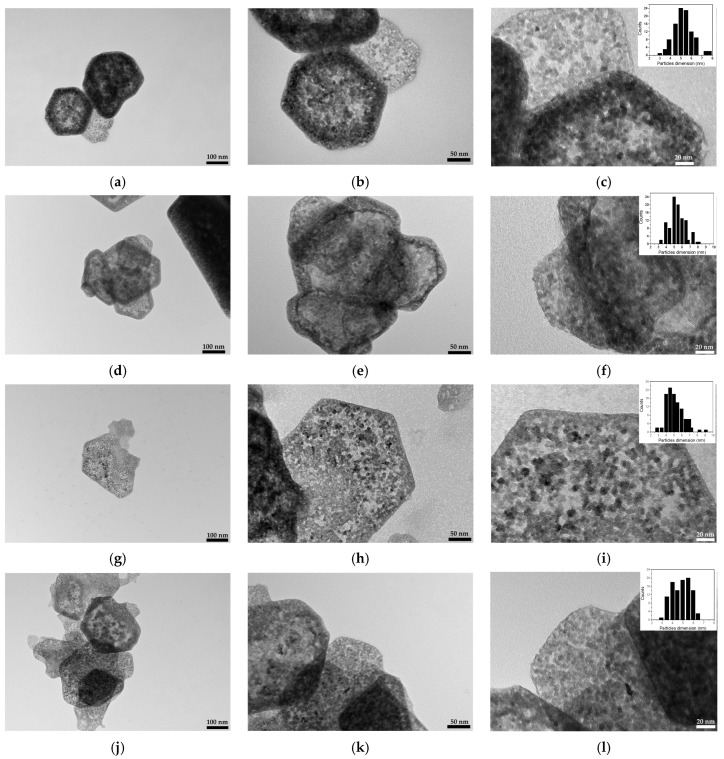
TEM images of the SK samples obtained by using the 3*R* ratio but varying the synthesis temperature. (**a**–**c**) The *3R_T 8 °C* sample; (**d**–**f**); the *3R_T 18 °C* sample; (**g**–**i**) the *3R* sample; (**j**–**l**) the *3R_T 45 °C* sample. The inset reported in (**c**,**f**,**j**,**l**) refers to the particles’ size distribution.

The dimensions and morphologies of the produced SK NPS were analyzed by using transmission electron microscopy (TEM). The collected TEM images revealed that in all of the considered samples, independently of the synthesis temperature, highly uniform, definite, and sharp hexagonal nanosheets were formed, typical of simonkolleite [11,23]. These hexagonal lamellae, characterized by a side dimension generally less than 100 nm and so thin that they are almost transparent in an electron beam, appeared to overlap each other and tended to aggregate along the basal plane (Figure 6a,d,g,j).

At higher magnification (Figure 6b,e,h,k), each lamella was composed of an ordered assembly of very small monodispersed primary nanoparticles (*singlets*), with dimensions in the range of 3–8 nm (inset of Figure 6c,f,i,l) and forming large mesoporous aggregates characterized by a definite hexagonal shape.

The quite uniform size distribution of the *singlets* can be attributed to the fast and diffuse nucleation occurring when the synthesis process starts, giving rise to the formation of solid primary NPs at the same time and under the same conditions during the reaction. Moreover, the fast kinetics of the ion exchange process (see Table 2) leads to the very rapid extinction of the OH^−^ sites on the resin substrate, preventing the growth of the formed primary NPs.

In particular, when lower synthesis temperatures were employed (*3R_T 8 °C* and *3R_T 18 °C* samples), a denser mesoporous hexagonal structure was noted (Figure 6a–d), and each lamella appeared to be composed of a *singlet* distribution mainly centered at around 5–6 nm (inset of Figure 6c–f). Concerning the *3R* and *3R_T 45 °C* samples (Figure 6g–j), a less dense mesoporous structure can be recognized and the hexagonal nanosheets are formed via the self-assembly of solid primary NPs of about 4–5 nm (inset of Figure 6i–l).

**Figure 7:**
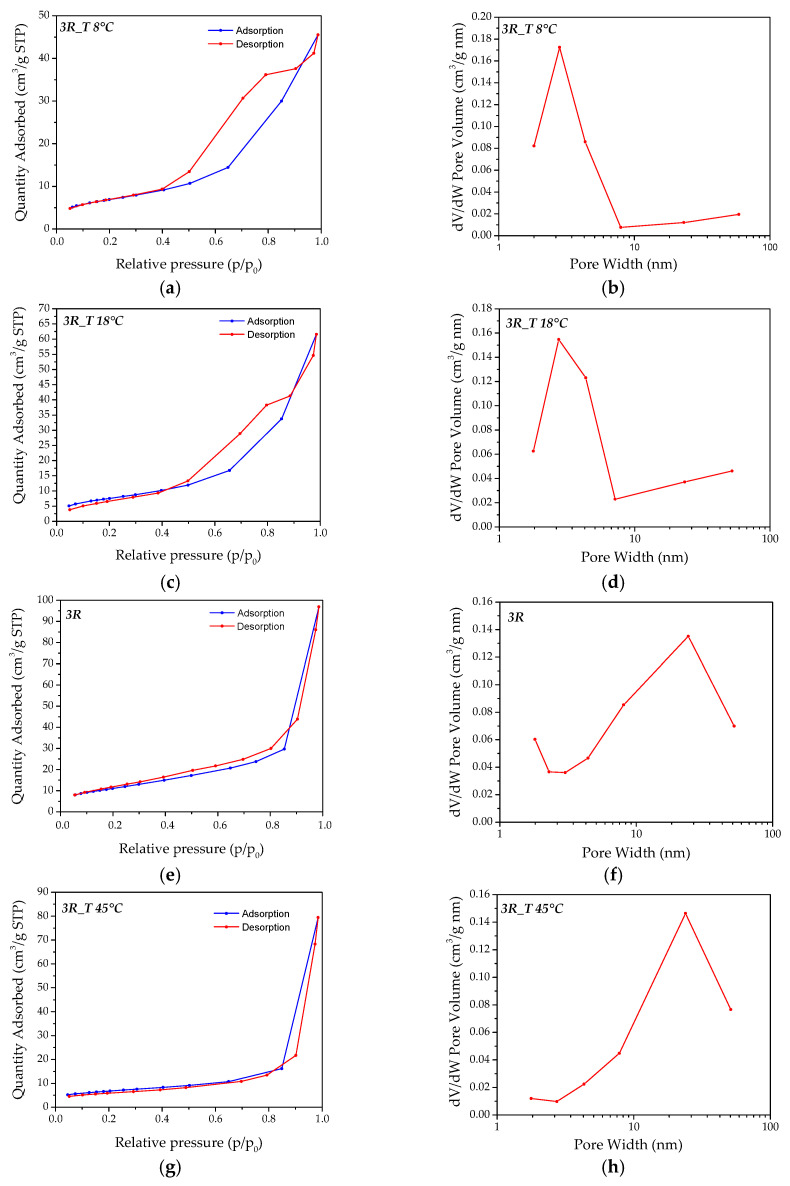
N_2_ absorption/desorption isotherms and pore size distribution of SK samples obtained by varying synthesis temperature. (**a**,**b**) *3R_T 8 °C* sample; (**c**,**d**); *3R_T 18 °C* sample; (**e**,**f**) *3R* sample; (**g**,**h**) *3R_T 45 °C* sample.

The formation of a mesoporous structure, observed in the TEM investigations, was confirmed by using the Brunauer–Emmett–Teller (BET) method (Figure 7). In particular, both the pore size distribution, obtained by using the Barrett–Joyner–Halenda (BJH) method from the desorption branch of the isotherm, and the physical adsorption/desorption results of N_2_ at 77K, referring to the *3R_T 8 °C*, *3R_T 18 °C*, *3R*, and *3R_T 45 °C* samples, are reported in Figure 7a–h, respectively.

According to the IUPAC classification, the adsorption isotherm of all of the considered samples can be classed as type IV (Figure 7a,c,e,g), with the H3 type hysteresis typical of mesoporous materials, which is associated with capillary condensation taking place in mesopores and revealing the presence of non-rigid aggregates of plate-like particles forming slit-like pores [38,39]. The presence of a mesoporous structure, in agreement with the type IV adsorption isotherm, was confirmed by the BJH pore size distribution (Figure 7b,d,f,h), showing the formation of mesopores in the 2–50 nm range [40]. In particular, the *3R_T 8 °C* and *3R_T 18 °C* samples were characterized by mesopores mainly peaking at around 3 nm (Figure 7b–d), smaller dimensions than those observed for the samples coming from higher synthesis temperatures (Figure 7f–h). This result confirms the formation of a denser mesoporous structure, in accordance with the TEM observations.

Concerning the BET specific surface areas, all of the samples exhibited values comparable to or even larger than those reported in literature works on simonkolleite-based materials [12,41]. In particular, by increasing the synthesis temperatures, the BET values gradually increased, ranging from about 25 m^2^/g to 41 m^2^/g (Table 3). The lowest BET value observed for the sample synthesized at T = 45° C (*3R_T 45 °C* sample) can be explained considering that, as also confirmed by XRD, at higher temperatures, the kinetics of SK formation was very rapid, leading to the production of a high quantity of pure SK in just 1 min. As we stopped this synthesis after 2 min to increase CC reduction, the produced NPs tended to agglomerate, also probably thanks to the high temperature conditions.

In conclusion, considering that SK NPs are generally produced by means of not environmentally friendly processes [15,30,32,33], lacking the fundamental aspect of sustainability, the possibility offered by our synthesis method to obtain up to almost 10 kg/week of pure and crystalline SK NPs in only 8 min, working in water, under ambient conditions of pressure/temperature, with low energy consumption and without the production of any damaging waste, constitutes an important milestone in terms of the sustainability and scalability of the process itself.

In addition, the possibility to produce simonkolleite NPs, acting as precursors to ZnO nanosheets at ambient temperature, supports the sustainability of the process considering that the conversion of simonkolleite into ZnO nanosheets generally occurred via annealing at higher temperatures [16,42].

## 4. Conclusions

The innovative and sustainable synthesis method presented in this study allows for the efficient production of pure simonkolleite nanoparticles in a rapid, environmentally friendly, and cost-effective manner. The process operates in water, under ambient conditions, with low energy consumption, and avoids the use of toxic reagents. The short synthesis times (only 8 min), the high production yield, and the scalability (almost 10 kg NPs/week), combined with the mesoporous structure (slit-like pores with dimensions in the 2–50 nm range) and promising surface area of the produced simonkolleite nanoparticles (up to 41 m^2^/g), highlight the potential of this approach for large-scale applications. The produced SK NPs appeared to be pure and crystalline, characterized by a highly uniform hexagonal lamellar with each nanosheet composed of an ordered assembly of very small monodispersed primary NPs with dimensions in the range of 3–8 nm.

Additionally, the ability to convert simonkolleite nanoparticles into ZnO nanosheets at room temperature further enhances the sustainability of the process, positioning it as a viable alternative for industrial applications in various fields, including health, chemistry, and cosmetics.

## Figures and Tables

**Table 1:** CC values and corresponding Y at different times (t) from beginning to end of synthesis. Values refer to syntheses performed at ambient temperature (T = 25 °C) by varying resin/ZnCl_2_ (*R*) ratios.

t (Min)	*0.8R* Sample		*1R* Sample		*1.5R* Sample		*2R* Sample		*3R* Sample	
	CC (g/L)	Y (%)	CC (g/L)	Y (%)	CC (g/L)	Y (%)	CC (g/L)	Y (%)	CC (g/L)	Y (%)
0	12.1	-	12.1	-	12.1	-	12.1	-	12.1	-
0.5	5.88	51.4	4.31	64.4	4.09	66.2	4.08	66.3	2.59	78.6
1	5.56	54.0	4.30	64.5	4.07	66.4	3.98	67.1	2.22	81.6
2	5.53	54.3	4.29	64.5	4.02	66.8	3.82	68.4	2.07	82.9
3	5.41	55.3	4.20	65.3	3.92	67.6	3.79	68.7	1.65	86.4
4	5.29	56.3	4.06	66.5	3.81	68.5	3.63	70.0	1.38	88.6
5	5.15	57.4	4.01	66.9	3.71	69.4	3.45	71.5	1.16	90.4
6	4.97	58.9	3.87	68.0	3.47	71.3	3.04	74.9	0.92	92.4
7	4.96	59.0	3.68	69.6	3.31	72.6	3.00	75.2	0.64	94.7
8	4.93	59.3	3.66	69.7	3.11	74.3	2.97	75.5	0.25	97.9

**Table 2:** The CC values and the corresponding Y at different times (t) from the beginning to the end of synthesis. The values refer to the syntheses performed by using the *3R* ratio but considering different temperature conditions.

t (Min)	*3R_T 8 °C* Sample		*3R_T 18 °C* Sample		*3R* Sample		*3R_T 45 °C* Sample	
	CC (g/L)	Y (%)	CC (g/L)	Y (%)	CC (g/L)	Y (%)	CC (g/L)	Y (%)
0	12.1	-	12.1	-	12.1	-	12.1	-
0.5	5.87	51.5	5.19	57.1	2.59	78.6	2.79	76.9
1	5.51	54.5	5.06	58.2	2.22	81.6	2.18	82.0
2	4.65	61.6	4.65	61.6	2.07	82.9	1.66	86.3
3	4.14	65.8	4.18	65.4	1.65	86.4	0.38	96.9
4	3.62	70.1	3.59	70.3	1.38	88.6	0.26	97.8
5	2.70	77.7	2.84	76.5	1.16	90.4	0.10	99.2
6	2.10	82.6	2.04	83.1	0.92	92.4	0.04	99.7
7	1.29	89.3	1.26	89.6	0.64	94.7	0.02	99.8
8	0.23	98.1	0.27	97.8	0.25	97.9	0.01	99.9

**Table 3:** The specific surface areas obtained by the BET equation fit for the N_2_ gas adsorption isotherms of the *3R_T 8 °C*, *3R_T 18 °C*, *3R*, and *3R_T 45 °C* samples. The pore volume from the BJH analysis is reported as well.

Sample	BJH Pore Volume (cc/g)	BET Surface Area (m^2^/g)
*3R_T 8 °C*	0.080	25.32
*3R_T 18 °C*	0.105	28.00
*3R*	0.140	40.65
*3R_T 45 °C*	0.120	24.02

## Data Availability

Data are contained within the article and Supplementary Materials.

## Supplementary Materials

The following supporting information can be downloaded at: https://www.mdpi.com/article/10.3390/nano14242005/s1, Figure S1. ICSD standard pattern for simonkolleite (Zn_5_Cl_2_(OH)_8_·H_2_O, ICSD #98-003-4904).
