# Supplementary material for: Innovative One-Step Sustainable Process to Produce Simonkolleite Nanoparticles
Source: Nanomaterials (Basel). 2024 Dec 13;14(24):2005. doi: 10.3390/nano14242005 (PMC11728450; doi:10.3390/nano14242005)
Supplement: Supplementary file 1 [file nanomaterials-14-02005-s001.zip › nanomaterials-3343289-supplementary.pdf]

Supplementary Material

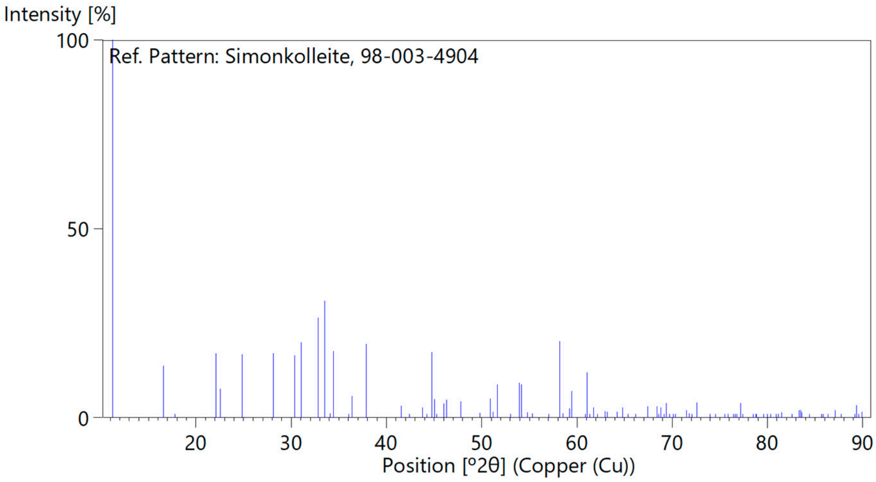

Figure S1. ICSD standard pattern for simonkolleite ($Zn_5Cl_2(OH)_8{\cdot}H_2O$, ICSD #98-003-4904).
